# Nervonic acid level in cerebrospinal fluid is a candidate biomarker for depressive and manic symptoms: A pilot study

**DOI:** 10.1002/brb3.2075

**Published:** 2021-02-18

**Authors:** Yuki Kageyama, Yasuhiko Deguchi, Kotaro Hattori, Sumiko Yoshida, Yu‐ichi Goto, Koki Inoue, Tadafumi Kato

**Affiliations:** ^1^ Department of Psychiatry, and Sackler Institute for Developmental Psychobiology Weill Cornell Medicine Brain and Mind Research Institute New York NY USA; ^2^ Department of Neuropsychiatry Graduate School of Medicine Osaka City University Osaka Japan; ^3^ Laboratory for Molecular Dynamics of Mental Disorders RIKEN Center for Brain Science Saitama Japan; ^4^ Medical Genome Center National Center of Neurology and Psychiatry Tokyo Japan; ^5^ Department of Psychiatry National Center of Neurology and Psychiatry Hospital Tokyo Japan; ^6^ Department of Psychiatry and Behavioral Science Juntendo Graduate School of Medicine Tokyo Japan

**Keywords:** biomarker, bipolar disorder, cerebrospinal fluid, major depressive disorder, nervonic acid

## Abstract

**Objective:**

Our previous metabolomics study showed that the plasma nervonic acid levels were higher in patients with major depressive disorder (MDD) than those in healthy controls and patients with bipolar disorder (BD). To examine whether the nervonic acid levels differ in the central nervous system, we investigated the levels in the cerebrospinal fluid (CSF) of patients with MDD, BD, and healthy controls.

**Methods:**

Nervonic acid levels in CSF were measured by gas chromatography time‐of‐flight mass spectrometry. The participants included 30 patients with MDD, 30 patients with BD, and 30 healthy controls.

**Results:**

In contrast to our previous study, no significant differences were found in the nervonic acid level in the CSF among the patients with MDD, BD, and the healthy controls. Though no significant state‐dependent changes were found among the three groups, we did observe a significant negative correlation between the nervonic acid levels and depressive symptoms in the depressive state of patients with MDD and BD (r = −0.38, *p* = .046). Further, a significant positive correlation was found between the nervonic acid levels and manic symptoms in the manic state of patients with BD (r = 0.79, *p* = .031).

**Conclusion:**

The nervonic acid levels in the CSF did not differ among the patients with MDD, BD, and the healthy controls; however, a significant negative correlation with depressive symptoms and a positive correlation with manic symptoms was observed. Thus, the nervonic acid levels in the CSF may be a candidate biomarker for mood symptoms.

## Graphical abstract

The cerebrospinal fluid (CSF) nervonic acid levels were not different among major depressive disorder patients (MDD), bipolar disorder patients (BD), and healthy controls.CSF nervonic acid levels had significant negative correlation with depressive symptom in MDD and BD, and positive correlation with manic symptom in BD.Further study is needed to understand the biological meaning of CSF nervonic acid level in MDD and BD.

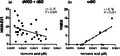

## 1 INTRODUCTION

Mood disorders are mainly diagnosed by self‐reported symptoms and clinical interviews. Currently, major depressive disorder (MDD) cannot be distinguished from the first depressive episode of bipolar disorder (BD) without a history of (hypo)mania. Previous reports showed that it takes 8–10 years from the onset of BD for the initiation of maintenance treatment (Baldessarini et al., 2003; Drancourt et al., 2013). This leads to worsening of a patient's social prognosis during the undiagnosed period. To solve this problem, a clinically applicable biomarker that distinguishes MDD from BD is required. We previously showed that plasma nervonic acid levels are increased in patients with MDD compared with nervonic acid levels in patients with BD and healthy controls (Kageyama et al., 2018). Therefore, the next step in biomarker research is to elucidate the relationship between depression pathophysiology and individual markers to help refine the current MDD criteria for diagnosis (Young et al., 2016).

Nervonic acid (IUPAC name: (*Z*)‐Tetracos‐15‐enoic acid) is a long chain of monounsaturated omega‐9 fatty acid. It is essential for the growth and maintenance of the brain and peripheral nervous tissue enriched in sphingomyelin (Martínez & Mougan, 2002). Because sphingomyelin is a key constituent of myelin, nervonic acid is abundant in the white matter of the brain (Li et al., 2019). Further, sphingomyelin is also associated with the regulation of neurogenesis. Previous studies reported that there is an impaired integrity of the white matter in patients with MDD (Li et al., 2007; Nobuhara et al., 2006; Velzen et al., 2020). Thus, a dysregulation of sphingomyelin metabolic pathway and decreased neurogenesis in the hippocampus has been suggested to play a role in depression (Gulbins et al., 2015). Antidepressants enhance the neurogenesis by inhibiting a part of the sphingomyelin metabolic pathway such as the acid sphingomyelinase/ceramide system (Gulbins et al., 2013). Moreover, the effects of lipid rafts on neurotransmitter signaling are also implicated in neurological and psychiatric diseases (Allen et al., 2007). Thus, the increased plasma nervonic acid levels might reflect the dysregulation of oligodendrocytes, sphingomyelin‐rich lipid rafts, and sphingomyelin metabolic pathway in patients with MDD. Although blood fatty acid composition and erythrocyte membrane phospholipids are known accessible indicators of fatty acids in the nervous system, neuronal membranes, and brain phospholipids (Assies et al., 2001; Babin et al., 1993; Song et al., 2018), it is still unclear whether the nervonic acid level in the central nervous system differs among the patients with MDD, BD, and the healthy controls.

Thus, we examined whether the nervonic acid levels in the cerebrospinal fluid (CSF) differ in patients with MDD, BD, and the healthy controls using gas chromatography time‐of‐flight mass spectrometry (GC‐TOFMS**)**.

## 2 MATERIALS AND METHODS

### 2.1 Participants

The CSF samples (30 samples from patients with MDD, 30 from patients with BD, and 30 from healthy controls) were derived from the National Center of Neurology and Psychiatry (NCNP) Biobank (project number: NCNPBB‐0050). The patients and healthy controls were either recruited at the NCNP Hospital or through advertisements on websites and in free local magazines. Trained psychologists and psychiatrists conducted a structured interview for all the participants by using a Japanese version of the Mini‐International Neuropsychiatric Interview (M.I.N.I.) (Sheehan et al., 1998). Then, diagnoses were made according to the criteria in Diagnostic and Statistical Manual of Mental Disorders, 4th edition, Text Revision (DSM‐IV‐TR) (American Psychiatric Association, 1994). Patients with comorbid axis I disorders, severe head trauma, substance abuse, substance dependence, or a prior history of central nervous system diseases, were excluded. The depressive symptoms were assessed using a Japanese version of the 21‐item Hamilton Depression Rating Scale (HAMD‐21). (Hamilton, 1960) Finally, the Young Mania Rating Scale (YMRS) (Young et al., 1978) was used to measure the severity of the manic episodes in patients with BD.

### 2.2 Ethics statement

All participants provided their written informed consent after the study procedures had been fully explained. The patient's records and information were anonymized and de‐identified before the analysis. This study was conducted in accordance with the Declaration of Helsinki and approved by the Ethics Committee of Osaka City University (approval number: 3886) and the Ethics Committee of NCNP (approval number: A2018‐007).

### 2.3 GC‐TOFMS analysis

For the GC‐TOFMS analysis, we followed the method described in our previous publication (Kageyama et al., 2018). In brief, 100 µl of CSF samples was added to 25 µl of 1N HCL, 400 µl of methanol, and 1,000 µl of isooctane. Then, 2.5 ng of oleic acid‐d9 (Avanti Polar Lipids, AL, USA) was added to each CSF sample as an internal standard. The solution was thoroughly mixed and centrifuged at 800 × *g* for 2 min at room temperature. The supernatant was evaporated and then dissolved in 40 µl of pyridine, 40 µl of *N*‐(*tert*‐Butyldimethylsilyl)‐*N*‐methyltrifluoroacetamide, and 30 µl of acetonitrile for the GC‐TOFMS analysis by using JMS‐T100GCV (JEOL). The absolute concentration was quantified using a standard reference of nervonic acid (Sigma‐Aldrich).

### 2.4 Statistical analysis

The data are reported as means ± standard deviation (*SD*). The means were compared using a Welch *t* test and one‐way analysis of variance with *post hoc* Tukey's test. The categorical variables were compared using the Chi‐squared test, and the outliers were determined by Smirnov–Grubbs' test. The results for independent variables were typically reported as odds ratios with 95% confidence intervals, and the Spearman's rank correlation coefficient was used for correlation analysis. The threshold for the statistical analyses was set at *p* < .05, and multiple comparisons were performed by estimating the false discovery rate using *q*‐values. The statistical power was calculated using G‐Power version 3.1 (Faul et al., 2007) where we regarded the value of power (1 –β) as 0.80. Finally, these statistical analyses were performed using the Graph‐Pad Prism 8.3.1 statistical program (GraphPad Software Inc.) and R version 3.6.3 (http://cran.r‐project.org/).

## 3 RESULTS

### 3.1 Characteristics of the participants

The demographic and clinical data of the participants are shown in Table 1. There were no significant differences in the sex ratios and ages among the groups.

**TABLE 1:** The demographic and clinical data of the participants

	MDD	BD	Control	Statistics
Number (male/female)	30 (15/15)	30 (13/17)	30 (15/15)	*p* =.62 ^a^
Age in years (mean ± *SD*)	43.8 ± 14.2	43.4 ± 11.8	43.1 ± 11.7	*p* =.98 ^b^
HAMD−21 score (mean ± *SD*)	12.3 ± 8.9	10.8 ± 8.4	*N*.A.	*p* =.52 ^c^
YMRS score (mean ± *SD*)	*N*.A.	4.1 ± 5.9	*N*.A.	*N*.A.

Abbreviations: BD, bipolar disorder; HAMD‐21, 21‐item Hamilton Depression Rating Scale; MDD, major depressive disorder; *N*.A., not applicable; *SD*, standard deviation; YMRS, Young Mania Rating Scale.

^a^ Chi‐squared test.

^b^ Analysis of variance.

^c^ Welch *t* test.

### 3.2 GC‐TOFMS analysis

**FIGURE 1:**
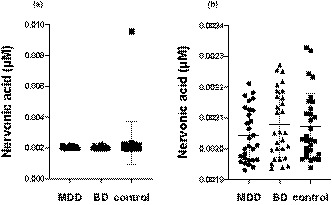
The nervonic acid level of cerebrospinal fluid in the three groups. The bars indicate the mean of each group. Abbreviations: BD, bipolar disorder; MDD, major depressive disorder

We obtained the absolute levels of nervonic acid in CSF in 88 of 90 samples. The nervonic acid level could not be detected in the remaining two participants (MDD, *n* = 1; BD, *n* = 1). There were no significant differences observed among the level of nervonic acid in the CSF of patients with MDD, BD, and the control group (MDD, 0.0020 ± 0.00086 µM; BD, 0.0021 ± 0.00011 µM; control, 0.0023 ± 0.0014 µM; *p* =.39; Figure 1a). The effect size was *f* = 0.20.

Since the highest value in the control group was statistically rejected by the Smirnov–Grubbs test (*p* = 2.2*e* ^−16^), we omitted that data. After omitting the outlier, there were no significant differences among the levels of nervonic acid in the CSF of patients with MDD, BD, and the control group (MDD, 0.0020 ± 0.00086 µM; BD, 0.0021 ± 0.00011 µM; control, 0.0021 ± 0.00011 µM; *p* =.36; Figure 1b). The effect size was *f* = 0.21.

### 3.3 Comparison of the nervonic acid levels in the CSF in a state‐dependent manner in patients with MDD and BD

**FIGURE 2:**
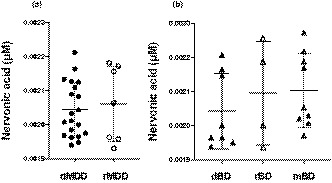
State‐dependent nervonic acid level in cerebrospinal fluid of patients with MDD and BD. The bars indicate the mean of each group. (a) Comparison between patients with dMDD and rMDD according to Welch *t* test. (b) Comparison among patients with dBD, rBD, and mBD according to one‐way ANOVA with *post hoc* Tukey's test. Abbreviations: MDD, major depressive disorder; BD, bipolar disorder; dMDD, depressive patients with major depressive disorder; rMDD, remitted patients with major depressive disorder; dBD, depressive patients with bipolar disorder; rBD, remitted patients with bipolar disorder; mBD, manic patients with bipolar disorder

To investigate whether the nervonic acid level showed a state‐dependent change in patients with MDD, we categorized them into two groups: depressed MDD (dMDD; *n* = 20) and remitted MDD (rMDD; *n* = 7), according to the results of M.I.N.I. (Table S1). The data of two patients with MDD were omitted because they had no M.I.N.I. data. We observed no significant difference in the nervonic acid levels between dMDD and rMDD patients (dMDD, 0.0020 ± 0.00081 µM; rMDD, 0.0021 ± 0.00042 µM; *p* =.72; Figure 2a). To investigate whether the nervonic acid level showed a state‐dependent change in patients with BD, we categorized them into three groups: depressed BD (dBD; *n* = 8), remitted BD (rBD; *n* = 4), and manic BD (mBD; *n* = 9), according to the results of M.I.N.I. (Table S2). The data of eight patients with BD were omitted because they had no M.I.N.I. data. There was no significant difference in the nervonic acid levels among patients with dBD, rBD, and mBD (dBD, 0.0020 ± 0.00011 µM; rBD, 0.0021 ± 0.00015 µM, mBD, 0.0021 ± 0.00011 µM; *p* =.55; Figure 2b). Then, we compared the nervonic acid levels in patients with dMDD and dBD (Table S3) and observed no significant difference between the two groups (*p* =.96). Finally, no significant difference was observed in the nervonic acid levels between the patients with rMDD and rBD as well (*p* =.71; Table S4).

### 3.4 Relationship between the nervonic acid levels in the CSF and the clinical assessments

**FIGURE 3:**
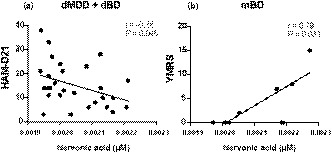
Scatter plots of the nervonic acid level in the cerebrospinal fluid versus the clinical symptoms in patients with MDD and BD. Each dot represents the data of one participant. (a) HAMD‐21 scores and nervonic acid level in patients with dMDD and dBD. (b) YMRS scores and nervonic acid level in patients with mBD. Abbreviations: HAMD‐21, 21‐item Hamilton Depression Rating Scale; dMDD, depressive state of major depressive disorder; dBD, depressive state of bipolar disorder, YMRS, Young Mania Rating Scale; mBD, manic state of bipolar disorder

To investigate the relationship between psychiatric symptoms (HAMD‐21 score for dMDD and dBD; YMRS score for mBD) and the nervonic acid levels in the CSF, we conducted a correlation analysis using Spearman's rank correlation coefficients. There was a significant negative correlation between the nervonic acid levels in patients with dMDD and dBD and their HAMD‐21 score (r = −0.38, *p* =.046; Figure 3a). The data of one patient out of nine patients with mBD were omitted because they had no YMRS data. There was a significant positive correlation between the nervonic acid levels in patients with mBD and their YMRS scores (r = 0.79, *p* =.031; Figure 3b).

### 3.5 Results of medication on the nervonic acid levels

We suspected that an antidepressant or mood stabilizer might affect the nervonic acid levels of CSF. To confirm the effect of medication on nervonic acid levels, we categorized the patients with MDD into two subgroups: patients receiving an antidepressant (medicated patients; *n* = 25) and drug‐free patients (*n* = 5). There was no significant difference observed between the two groups (medicated, 0.0020 ± 0.000076 µM; drug‐free, 0.0021 ± 0.0012 µM; *p* =.32; Figure S1a). We also categorized the patients with BD into two subgroups: patients receiving a mood stabilizer (medicated patients; *n* = 26) and drug‐free patients (*n* = 4). There was no significant difference observed between these two groups as well (medicated, 0.0021 ± 0.00011 µM; drug‐free, 0.0020 ± 0.000081 µM; *p* =.12; Figure S1b).

## 4 DISCUSSION

To the best of our knowledge, this is the first study to investigate the nervonic acid level in the CSF among patients with MDD, BD, and healthy controls. In contrast to our hypothesis, there was no significant difference in the nervonic acid level in the CSF among these three groups. However, we observed a significant negative correlation of the nervonic acid level with depressive symptoms and a positive correlation with manic symptoms. These results indicate that the nervonic acid level in the CSF may be a candidate biomarker for evaluating depressive and manic symptoms in mood disorders. Yet, the sample size was too small to draw a conclusion.

### 4.1 A comparison with previous studies related to nervonic acid levels in mood disorders

In peripheral samples, the nervonic acid levels in the plasma and erythrocytes are significantly lower in patients with recurrent depression than in healthy people. (Assies et al., 2010) This was an inconsistent finding when compared to our previous results (Kageyama et al., 2018) and might be attributed to the difference in the type of sample used (erythrocyte versus plasma) or the subtypes of MDD (recurrent versus single episode) that were studied. (Hayashi‐Takagi et al., 2014) In the central nervous system (CNS) samples, a previous study showed that there were no differences in the nervonic acid levels in the prefrontal cortex and corpus callosum of patients with MDD, BD, schizophrenia, and healthy controls (Hamazaki et al., 2015, 2017); this is consistent with our results. Therefore, to compare the nervonic acid levels in the peripheral blood and CNS, we should consider the blood–brain barrier permeability and whether they are regulated by common biological processes. (Hayashi‐Takagi et al., 2014).

The synthesis of nervonic acid is regulated by *SCD1*, *SCD5*, *ELOVL1, ELOVL2, ELOVL3, ELOVL4, ELOVL5, ELOVL6,* and *ELOVL7*. (Castro et al., 2016; Miyazaki & Ntambi, 2008) According to the Genotype‐Tissue Expression database (Mele et al., 2015) (GTEx Portal v8; dbGaP accession: phs000424.v8.p2), the median expression of *SCD1*, *SCD*5, *ELOVL2*, *ELOVL4*, *ELOVL6*, and *ELOVL7* is higher in the brain than in the blood (Table S5); the expression of *ELOVL5* in the whole body, as per the GTEx project, is shown in the Figure S2. This suggested that the biological processes of the synthesis of nervonic acid may be different in the CNS and peripheral blood.

A sodium‐dependent membrane transporter transports docosahexaenoic acid, an essential omega‐3 fatty acid, into the brain. (Nguyen et al., 2014) Nervonic acid, an omega‐9 fatty acid, is supposedly transported through the blood–brain barrier, particularly in newborns. (Cook et al., 1998) The patients with MDD reportedly lose their blood–brain barrier integrity through the loss of the tight‐junction protein claudin‐5, a major cell adhesion molecule that forms a para‐cellular barrier between the endothelial cells. (Dudek et al., 2020; Kealy et al., 2020) This blood–brain barrier dysfunction leads to the infiltration of peripheral substances. Thus, these two factors may allow nervonic acid to cross the blood–brain barrier; however, the dynamics of nervonic acid transport through the blood–brain barrier is still unclear.

By taking the biological processes and blood–brain barrier permeability of the nervonic acid into consideration, it may be difficult to compare the nervonic acid levels in the peripheral blood and CSF. Although a significant difference was observed in the plasma nervonic acid level of the patients with MDD and healthy controls, we observed no such significant difference in the nervonic acid levels in the CSF; this might suggest that the biological interpretation of the nervonic acid level is different between the peripheral and CNS samples.

### 4.2 Nervonic acid level in the CSF and severity of depressive symptoms

The expression levels of *SCD* and *ELOVL5* in the postmortem prefrontal cortex are reportedly lower in patients with MDD than in the healthy controls. (Lalovic et al., 2010; McNamara & Liu, 2011) The *ELOVL5* gene is expressed in a region‐specific (Figure S2) (Mele et al., 2015) and a cell type‐specific (Medina & Tabernero, 2002) manner in the CNS. This leads to a region‐specific and cell type‐specific profile of fatty acid synthesis and incorporation into complex lipids, which may be related to depressive symptoms. (Müller et al., 2015) This suggests that the effects of expression change of *ELOVL5* may attribute to the negative correlation between the nervonic acid level in the CSF and the severity of the depressive symptoms in mood disorders; therefore, a further understanding of the lipid profiles in the CNS is important. (Deák et al., 2019).

### 4.3 Nervonic acid level in CSF and the severity of manic symptoms

The severity of manic symptoms is reported to be negatively correlated with the levels of arachidonic acid (20:4n‐6) and eicosapentaenoic acid (20:5n‐3), and positively correlated with the arachidonic acid: eicosapentaenoic acid ratio. (Sublette et al., 2007) Hence, the long‐chain unsaturated fatty acid level in the CNS may play a role in the severity of the manic symptoms; however, the precise mechanism is not known.

In this study, four mBD patients showed a YMRS score of 0, indicating that the manic symptoms had been ameliorated at the time of sampling. However, it is difficult to perform a CSF sampling in excited manic patients. This is a limitation of the study because of which we need to carefully evaluate the meaning of the positive correlation between the nervonic acid level and the severity of the manic symptoms.

This study has several other limitations as well. First, we did not evaluate the effect of the socioeconomic status, BMI levels, and physical illness of the patients on the nervonic acid level in the CSF. Second, the transport system of nervonic acid between the peripheral blood, brain, and CSF remains unclear. Third, the metabolic homeostasis of nervonic acid is not known. Lastly, the sample size was too small. Larger samples are needed to draw a definitive conclusion.

In conclusion, the nervonic acid level did not differ among the patients with MDD, BD, and healthy controls. Moreover, the nervonic acid level in the CSF had a significant negative correlation with depressive symptoms and a positive correlation with manic symptoms. Therefore, further studies are needed to understand the biological meaning of nervonic acid level in the CSF in patients with MDD and BD.

## CONFLICTS OF INTEREST

The authors declare no conflicts of interest.

## AUTHOR CONTRIBUTIONS

YK and YD conceived and designed the study and wrote the manuscript. YK acquired and analyzed the data. KH, SY, and YG collected the samples. TK supervised the project. All authors drafted and approved the final manuscript.

### PEER REVIEW

The peer review history for this article is available at https://publons.com/publon/10.1002/brb3.2075.

## Supporting information

Supplementary Material

## Data Availability

The data that support the findings of this study are available from the corresponding author upon reasonable request.
