# Supplementary material for: Nervonic acid level in cerebrospinal fluid is a candidate biomarker for depressive and manic symptoms: A pilot study
Source: Brain Behav. 2021 Feb 18;11(4):e02075. doi: 10.1002/brb3.2075 (PMC8035447; doi:10.1002/brb3.2075)
Supplement: Supplementary file 1 — Supplementary Material [file BRB3-11-e02075-s001.docx]

**Nervonic acid level in cerebrospinal fluid is a candidate biomarker for depressive and manic symptoms: a pilot study**

**Supplementary Information**

**Yuki Kageyama, Yasuhiko Deguchi, Kotaro Hattori, Sumiko Yoshida, Yu-ichi Goto, Koki Inoue, and Tadafumi Kato**

**Supplementary Table S1.** **The** **demographic and clinical data of patients with major depressive disorder**

|  | dMDD | rMDD | Statistics |
| --- | --- | --- | --- |
| Number (male/female) | 20 (9/11) | 7 (4/3) | *P* = 0.58 ^a^ |
| Age in years (mean ± SD) | 44.3 ± 14.6 | 42.9 ± 11.0 | *P* = 0.80 ^b^ |
| HAMD-21 score (mean ± SD) | 14.9 ± 8.8 | 5.7 ± 4.4 | *P* = 0.0028 ^b^ |

^a^ Chi-squared test

^b^ Welch-*t* test

Abbreviations: dMDD, depressed patients with major depressive disorder; rMDD, remitted patients with major depressive disorder; HAMD-21, 21-item Hamilton Depression Rating Scale; SD, standard deviation.

**Supplementary Table S2.** **The** **demographic and clinical data of patients with bipolar disorder**

|  | dBD | rBD | mBD | Statistics |
| --- | --- | --- | --- | --- |
| Number (male/female) | 8 (3/5) | 4 (2/2) | 9 (3/6) | *P* = 0.85 ^a^ |
| Age in years (mean ± SD) | 49.0 ± 12.7 | 45.0 ± 8.6 | 38.4 ± 8.5 | *P* = 0.17 ^b^ |
| HAMD-21 score (mean ± SD) | 15.6 ± 9.0 | 6.0 ± 1.6 | 7.6 ± 5.3 | *P* = 0.056 ^b^ |
| YMRS score (mean ± SD) | 0.88 ± 1.5 | 3.3 ± 2.9 | 4.0 ± 5.2 | *P* = 0.29 ^b^ |

^a^ Chi-squared test

^b^ Analysis of variance

Abbreviations: dBD, depressed patients with bipolar disorder; rBD, remitted patients with bipolar disorder; mBD, manic patients with bipolar disorder; HAMD-21, 21-item Hamilton Depression Rating Scale; YMRS, Young Mania Rating Scale; SD, standard deviation.

**Supplementary Table S3.** **The demographic and clinical data of the depressed patients with major depressive disorder and bipolar disorder**

|  | dMDD | dBD | Statistics |
| --- | --- | --- | --- |
| Number (male/female) | 20 (9/11) | 8 (3/5) | *P* = 0.72 ^a^ |
| Age in years (mean ± SD) | 44.3 ± 14.6 | 49.0 ± 12.7 | *P* = 0.43 ^b^ |
| HAMD-21 score (mean ± SD) | 14.9 ± 8.8 | 15.6 ± 9.0 | *P* = 0.86 ^b^ |

^a^ Chi-squared test

^b^ Welch-*t* test

Abbreviations: dMDD, depressed patients with major depressive disorder; dBD, depressed patients with bipolar disorder; HAMD-21, 21-item Hamilton Depression Rating Scale; SD, standard deviation.

**Supplementary Table S4.** **The demographic and clinical data of the remitted patients with major depressive disorder and bipolar disorder**

|  | rMDD | rBD | Statistics |
| --- | --- | --- | --- |
| Number (male/female) | 7 (4/3) | 4 (2/2) | *P* = 0.82 ^a^ |
| Age in years (mean ± SD) | 42.9 ± 11.0 | 45.0 ± 8.6 | *P* = 0.78 ^b^ |
| HAMD-21, score (mean ± SD) | 5.7 ± 4.4 | 6.0 ± 1.6 | *P* = 0.89 ^b^ |

^a^ Chi-squared test

^b^ Welch-*t* test

Abbreviations: dMDD, depressed patients with major depressive disorder; dBD, depressed patients with bipolar disorder; HAMD-21, 21-item Hamilton Depression Rating Scale; SD, standard deviation.

**Supplementary Table S5. The expression of *SCD* and *ELOVL* genes in the brain and blood as obtained from the Genotype-Tissue Expression project**

|  | Brain-Cortex, n = 255 (Median TPM) | Whole Blood, n = 755 (Median TPM) |
| --- | --- | --- |
| *SCD1* | 8.15 | 0.03483 |
| *SCD5* | 131.2 | 0.3291 |
| *ELOVL1* | 23.09 | 43.84 |
| *ELOVL2* | 10.84 | 0.00848 |
| *ELOVL3* | N.A. | 0.8225 |
| *ELOVL4* | 11.49 | 0.1158 |
| *ELOVL5* | 26.12 | 39.86 |
| *ELOVL6* | 7.081 | 0.6895 |
| *ELOVL7* | 8.291 | 0.6278 |

Abbreviations: SCD, stearoyl-CoA desaturase; ELOVL, elongation of very long chain fatty acids; TPM, transcripts per million; N.A., not applicable.

**Supplementary Figure S1.** **Dot plots of the nervonic acid level in cerebrospinal fluid in patients with major depressive disorder and bipolar disorder.** The horizontal bars represent the mean values of the groups. (a) Comparison between the medicated patients with major depressive disorder (MDD) and drug-free patients with MDD using a Welch *t*-test (*P* = 0.32). (b) Comparison between the medicated patients with bipolar disorder (BD) and drug-free patients with BD using a Welch *t*-test (*P* = 0.12).

**
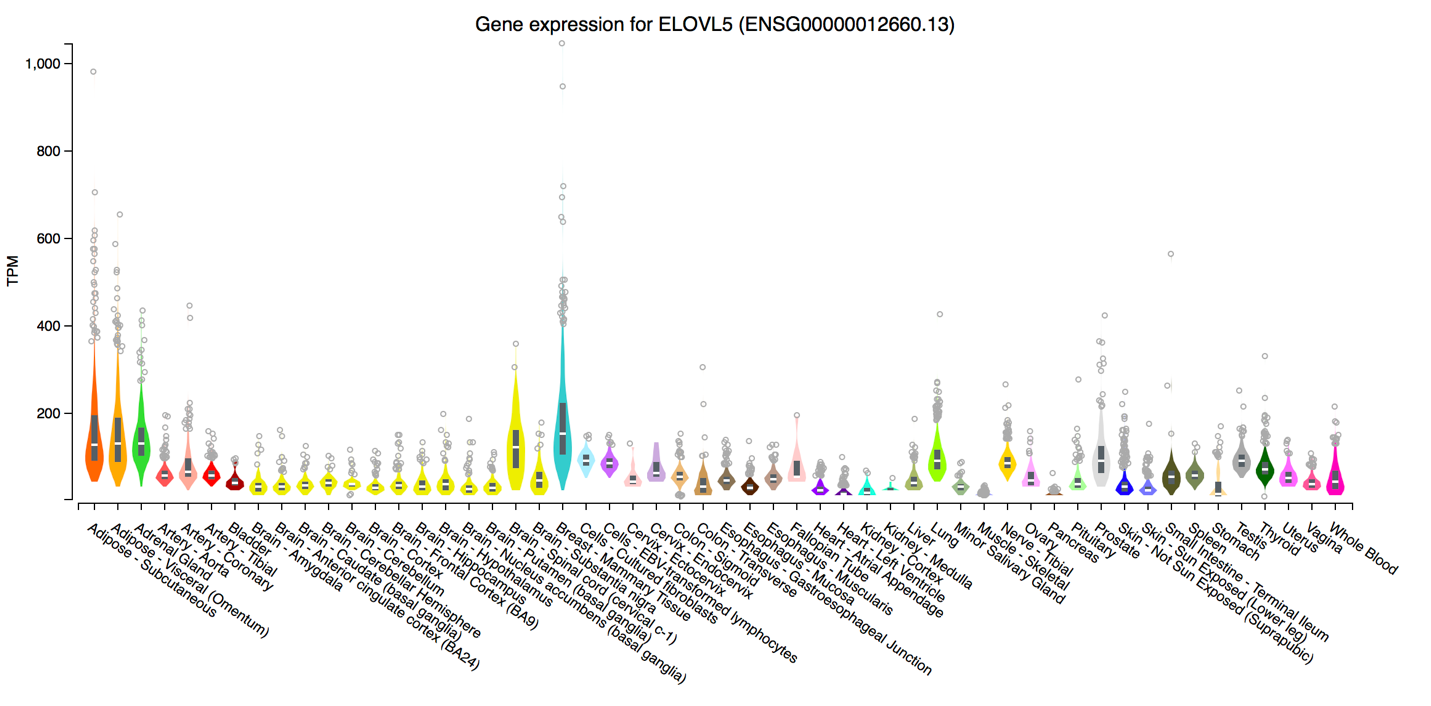
**

**Supplementary Figure S2.** **Expression of *ELOVL5* in the whole body; data obtained from the GTEx project**

Abbreviation: TPM, transcripts per million
